# Cramér–Rao Bounds for DoA Estimation of Sparse Bayesian Learning with the Laplace Prior

**DOI:** 10.3390/s23010307

**Published:** 2022-12-28

**Authors:** Hua Bai, Marco F. Duarte, Ramakrishna Janaswamy

**Affiliations:** Department of Electrical and Computer Engineering, University of Massachusetts, Amherst, MA 01003, USA

**Keywords:** Cramér–Rao lower bound, sparse Bayesian learning, Bayesian CRLB, Laplace prior, nonuniform array

## Abstract

In this paper, we derive the Cramér–Rao lower bounds (CRLB) for direction of arrival (DoA) estimation by using sparse Bayesian learning (SBL) and the Laplace prior. CRLB is a lower bound on the variance of the estimator, the change of CRLB can indicate the effect of the specific factor to the DoA estimator, and in this paper a Laplace prior and the three-stage framework are used for the DoA estimation. We derive the CRLBs under different scenarios: (i) if the unknown parameters consist of deterministic and random variables, a hybrid CRLB is derived; (ii) if all the unknown parameters are random, a Bayesian CRLB is derived, and the marginalized Bayesian CRLB is obtained by marginalizing out the nuisance parameter. We also derive the CRLBs of the hyperparameters involved in the three-stage model and explore the effect of multiple snapshots to the CRLBs. We compare the derived CRLBs of SBL, finding that the marginalized Bayesian CRLB is tighter than other CRLBs when SNR is low and the differences between CRLBs become smaller when SNR is high. We also study the relationship between the mean squared error of the source magnitudes and the CRLBs, including numerical simulation results with a variety of antenna configurations such as different numbers of receivers and different noise conditions.

## 1. Introduction

In signal processing, the direction of arrival (DoA) is a popular topic, and there exists a rich literature of estimation of the DoA of incoming sources from measurements of an antenna array. These approaches include the multiple signal classification (MUSIC) algorithm [1,2], estimation of signal parameters via rotational invariant techniques (ESPRIT) [3,4], and so on. These algorithms are applied for DoA estimation by partitioning vector space into a signal subspace and a noise subspace, with the signal subspace containing the incoming sources that we want to estimate in DoA. However, there exist several shortcomings or limitations when using the above algorithms. For example, when using the MUSIC algorithm, the number of signal source is needed as a priori knowledge, and the algorithm requires many measurements for the estimate of incoming sources [1].

Sparse Bayesian learning (SBL) [5] can be considered to overcome the limitations of mentioned DoA methods. SBL is also known as the relevance vector machine (RVM). It is a kind of technique for solving the regression problem under the Bayesian framework. Compared with a general least squares method [6], the sparsity of the solution is taken into account within SBL and the standard deviation of the result is also estimated in SBL. With the development of SBL, several methods which are based on SBL have been proposed [7,8,9] and applied to solve the DoA problem in [10,11,12,13,14]. In addition to overcoming the limitations of the mentioned traditional DoA methods and the estimation accuracy and efficiency are also improved according to simulation results, and the SBL-based method is also studied for the 2-D DoA problem in [15] and extended to the off-grid DoA estimation in [16]. The CRLB [17] of the regular SBL is studied and discussed in [18]. In order to further improve the performance of the estimation method, in [19,20,21,22], the Laplace prior is used in SBL instead of the Gaussian prior of the traditional SBL. Simulation results show that compared with the Gaussian prior, using the Laplace prior can obtain sparser estimation results. However, to the best of our knowledge, the CRLB of the Laplace prior-based DoA estimator has not been studied previously; it is the topic of this paper. In this paper, instead of proposing a new DoA estimation method we focus on deriving the lower bounds of the Laplace prior-based DoA estimator, the CRLBs are derived under different scenarios, different antenna configurations, different environmental noises, and different numbers of measurements. We present a comparison between the CRLBs and the estimator mean squared error (MSE) from numerical simulations, as well as a comparison between the CRLBs for the different situations.

This paper is organized as follows. In Section 2, the Laplace prior-based DoA estimation method used in this paper is introduced. In Section 3, the CRLBs under different situations are derived. Simulation results are presented in Section 4. The conclusion is provided in Section 5.

## 2. DOA via SBL with Laplace Prior

**Figure 1:**
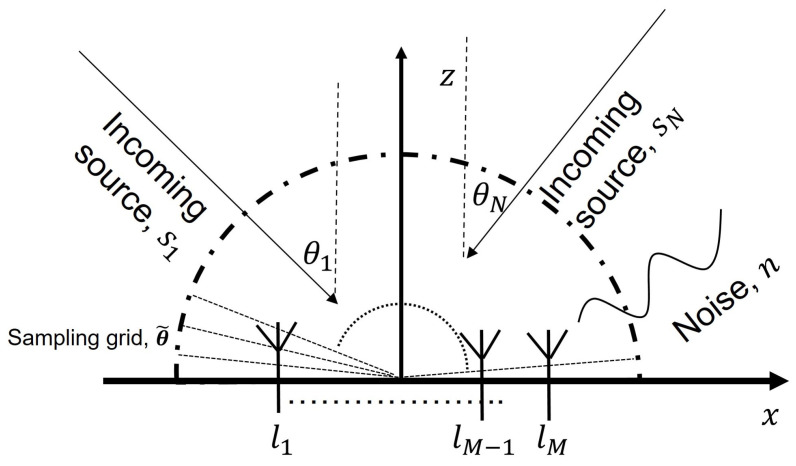
*M*-element nonuniform linear array.

In this paper, a nonuniform linear array, shown in Figure 1, is used as the receiver. The linear array is comprised of *M* identical elements with the *i*th element located at distance $l_{i}$ from the origin. The incoming sources are denoted by $s_{j}$ and the incident angles of the incoming sources are denoted by $\theta =\{\theta_{1},\ldots ,\theta_{N}\}$, where $\theta_{j}\in$ [−90°, 90°] and j=1,2,…,N. Independent complex white noise is also present in the element observations and denoted by $n_{i}$, i=1,…,M.

The received signal of the *i*th element, $y_{i}$, is written as

(1) $$\begin{array}{c} y_{i}=\sum_{j=1}^{N}s_{j}e^{-jkl_{i}\sin\theta_{j}}+n_{i},i=1,2,....,M, \end{array}$$

where *k* is the wave number. In matrix form (1) is written as

(2) $$\begin{array}{c} y=As+n, \end{array}$$

where $y={\left[y_{1}\;y_{2}\;\ldots \;y_{M}\right]}^{T}\in C^{M\times 1}$, $A\in C^{M\times N}$ is a matrix that encodes the antenna architecture into its entries $A\left(m,n\right)=e^{-jkl_{m}\sin\theta_{n}}$, $s={\left[s_{1}\;s_{2}\;\ldots \;s_{N}\right]}^{T}\in C^{N\times 1}$ and $n={\left[n_{1}\;n_{2}\;\ldots \;n_{M}\right]}^{T}\in C^{M\times 1}$; the latter vector n follows a circularly symmetric complex normal distribution with zero mean and covariance matrix $\Gamma =2\sigma^{2}I\in R^{M\times M}$, where $\sigma^{2}$ is usually unknown. In order to estimate the incoming source DoA, a DoA sampling grid $\tilde{\theta }=\left[{\tilde{\theta }}_{1}\;\ldots \;{\tilde{\theta }}_{K}\right]\in R^{K\times 1}$ is used where the source strength corresponding to $\tilde{\theta }$ is denoted by $\tilde{s}\in C^{K\times 1}$ and K>N. In this paper, we set the size of sampling grid K=181 to cover all integers from −90${}^{\circ }$ to 90${}^{\circ }$. Here $\tilde{A}\in C^{M\times K}$ is computed similarly to A but using all angles in the grid $\tilde{\theta }$. The real and imaginary part of the sources are separated by rewriting (2) as [11]

(3) $$\begin{array}{c} \bar{y}=\bar{A}\bar{s}+\bar{n}, \end{array}$$

where $\bar{y}=\left[\begin{array}{c} ℜ(y)\\ ℑ(y) \end{array}\right]$, $\bar{s}=\left[\begin{array}{c} ℜ(\tilde{s})\\ ℑ(\tilde{s}) \end{array}\right]$, $\bar{n}=\left[\begin{array}{c} ℜ(n)\\ ℑ(n) \end{array}\right]$ and $\bar{A}=\left[\begin{array}{cc} ℜ(\tilde{A}) & -ℑ(\tilde{A})\\ ℑ(\tilde{A}) & ℜ(\tilde{A}) \end{array}\right]$. By using the Laplace prior and adopting the multistage hierarchical model of [19], the DoA problem is converted to an $\ell_{1}$-norm regression problem and a sparser solution than that obtained by using the Gaussian prior can be obtained by solving the $\ell_{1}$-norm regression problem [23]. The multistage model used in this paper contains three stages. First,

(4) $$\begin{array}{c} P\left(\bar{s}|\Sigma \right)=N\left(\bar{s}|0,\Sigma \right), \end{array}$$

where *P* denotes probability, N(x|μ,Σ) denotes that x follows a multivariate Gaussian distribution with mean μ and covariance matrix Σ, $\Sigma =\mathrm{diag}\left(\alpha \right)=\mathrm{diag}(\alpha_{1},\alpha_{2}\cdots$$\alpha_{2K})$, and $\alpha_{i}$ denotes the variance for ${\bar{s}}_{i}$. Secondly, we have

(5) $$\begin{array}{c} P\left(\alpha_{i}|\lambda \right)=\frac{\lambda }{2}\exp\left(-\frac{\lambda \alpha_{i}}{2}\right),i=1,2,\ldots ,2K. \end{array}$$

Thirdly, Jeffrey’s hyperprior [24] is applied: $P\left(\lambda \right)\propto \frac{1}{\lambda }$. To estimate the unknown incoming sources, we first obtain estimates of α and λ by maximizing the marginal likelihood. The logarithm of marginal likelihood is written as [7]

(6) $$\begin{array}{cc} \log P(\bar{y}|\alpha ,\lambda ,\sigma^{2})= & -\frac{1}{2}\left[2M\log2\pi +\log|C|+{\bar{y}}^{T}C^{-1}\bar{y}\right]\\ \; & -\frac{\lambda }{2}\sum_{i=1}^{2K}\alpha_{i}+\left(2K-1\right)\log\lambda , \end{array}$$

where

(7) $$\begin{array}{c} C=\sigma^{2}I+\bar{A}\Sigma^{-1}{\bar{A}}^{T}. \end{array}$$

We can get the estimates for the parameters by using the conventional method of setting the derivative of the marginal likelihood with respect to the specific parameter to 0 [25], and solve for the parameters to obtain the estimates. Alternatively we can estimate the parameters using the “constructive” so-called fast marginal likelihood maximisation method [7], which is more efficient. The fast marginal likelihood maximisation method iterates its search for optimal values over the different parameters $\alpha_{i}$, optimizing one at a time. The first step of the *i*th iteration of the the method is to rewrite the term C in (7) as [7]

(8) $$\begin{array}{c} C=\sigma^{2}I+\sum_{j\neq i}\alpha_{j}^{-1}{\bar{A}}_{j}{\left({\bar{A}}_{j}\right)}^{T}+\alpha_{i}^{-1}{\bar{A}}_{i}{\left({\bar{A}}_{i}\right)}^{T}, \end{array}$$

where ${\bar{A}}_{i}$ denotes the *i*th column of $\bar{A}$. Then we can separate the term C into two parts, one is dependent on $\alpha_{i}$, which is equal to $\alpha_{i}^{-1}{\bar{A}}_{i}{\left({\bar{A}}_{i}\right)}^{T}$, and the other part is independent of $\alpha_{i}$ which is $C_{-i}\equiv \sigma^{2}I+\sum_{j\neq i}\alpha_{j}^{-1}{\bar{A}}_{j}{\left({\bar{A}}_{j}\right)}^{T}$. Then, we can similarly separate the marginal likelihood into two parts, the part which is dependent on $\alpha_{i}$ is [7]

(9) $$L\left(\alpha_{i}\right)=\frac{1}{2}\left[\log\frac{1}{1+\alpha_{i}p_{i}}+\frac{{\left(q_{i}\right)}^{2}\alpha_{i}}{1+\alpha_{i}p_{i}}-\lambda \alpha_{i}\right],$$

where $p_{i}={\left({\bar{A}}_{i}\right)}^{T}{\left(C_{-i}\right)}^{-1}{\bar{A}}_{i}$ and $q_{i}={\left({\bar{A}}_{i}\right)}^{T}{\left(C_{-i}\right)}^{-1}\left(\bar{y}\right)$. In the *i*th iteration, we only consider the maximum $L(\alpha_{i})$, and the optimal $\alpha_{i}$ that maximizes that partial marginal likelihood is estimated by [7]

(10) $$\alpha_{i}=\left\{\begin{array}{cc} \frac{-Y+\sqrt{Y^{2}-4XZ}}{2X}, & \mathrm{if}\;{\left(q_{i}\right)}^{2}-p_{i}>\lambda \\ 0, & \mathrm{if}\;{\left(q_{i}\right)}^{2}-p_{i}\leq \lambda , \end{array}\right.$$

where $X=\lambda p_{i}^{2}$, $Y=p_{i}^{2}+2\lambda p_{i}$, $Z=\lambda +p_{i}-q_{i}^{2}$. Once the marginal likelihood converges after iterating over all parameters or the specific requirements we set are satisfied such as the maximum iteration, we will stop the optimal parameter search. We use the estimated parameters and the posterior distribution over $\bar{s}$ to obtain the estimate for the incoming sources, the posterior distribution is

(11) $$\begin{array}{c} P\left(\bar{s}|\bar{y},\alpha ,\sigma^{2}\right)\propto P\left(\bar{y}|\bar{s},\sigma^{2}\right)P\left(\bar{s}|\Sigma \right)=N\left(\bar{s}|\bar{\mu },\bar{\Sigma }\right), \end{array}$$

where $\bar{\mu }$ and $\bar{\Sigma }$ are given by [5]

(12) $$\begin{array}{cc} \bar{\mu } & =\sigma^{-2}\bar{\Sigma }{\bar{A}}^{T}\bar{y}, \end{array}$$

(13) $$\begin{array}{cc} \bar{\Sigma } & ={\left(\Sigma^{-1}+{\bar{A}}^{T}\bar{A}/\sigma^{2}\right)}^{-1}. \end{array}$$

**Figure 2:**
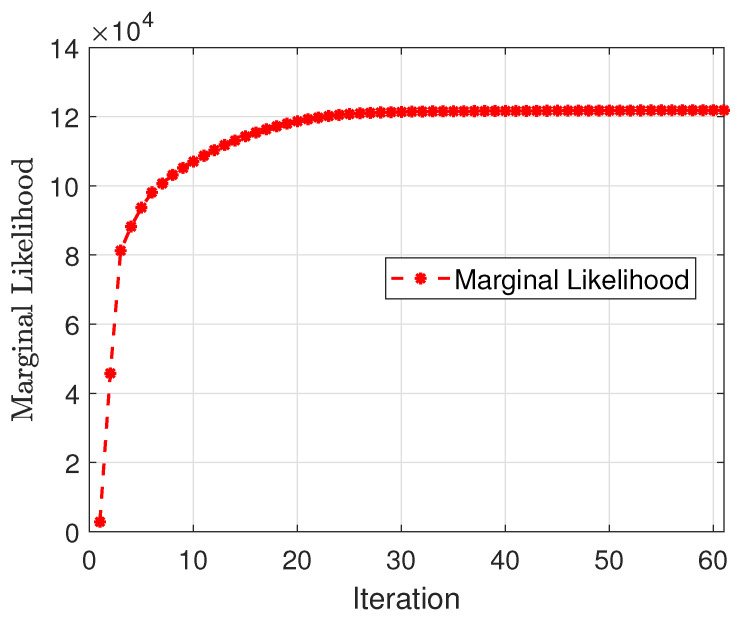
The change of marginal likelihood versus iteration.

**Figure 3:**
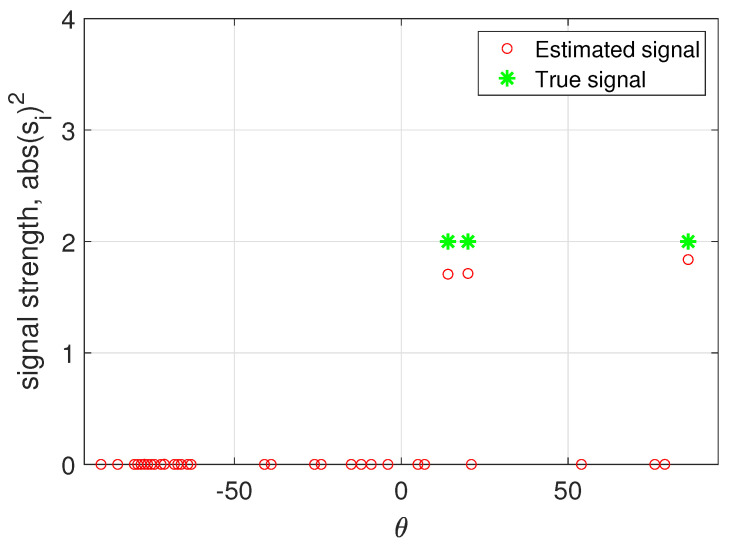
Estimated directions and strength of incoming signals.

In summary, we first update the unknown parameters used in the hierarchical model iteratively until the marginal likelihood converges, and then we use (12) and (13) to update the estimated mean and variance of the sources. Figure 2 presents the marginal likelihood versus iterations of one example and the estimated incoming signals are shown in Figure 3 with setting the convergence threshold as $10^{-6}$.

## 3. CRLBs for DoA Estimation with SBL with Laplace Prior

In this section, we will derive the CRLBs for the DoA estimator described in the previous section. There exist several types of CRLBs, and the appropriate CRLB should be used depending on the configuration of unknown parameters of the estimator [26,27,28]. The regular CRLB corresponds to the estimator in which all the unknown parameters are deterministic; the Bayesian CRLB corresponds to the estimator with random parameters, and the hybrid CRLB corresponds to the estimator with some random and some deterministic parameters. We want to use the derived CRLBs to study the effect of various factors in the performance of the DoA estimator, and we will compare the CRLBs under different situations. In principle, the DoA estimator in this paper works as a maximum likelihood estimator, and its performance can be measured by the MSE. We use the vector ϕ to denote the true value for the unknown variables. In this DoA problem, the unknown variables include the incoming source strengths, the noise, and the hyperparameters defined in the hierarchical model. We use $\bar{\phi }\left(y\right)$ to denote the estimate of ϕ from the available measurement y. The MSE of the estimated result is defined as

(14) $$\begin{array}{cc} \sigma_{\bar{\phi }} & =\frac{1}{n}\int \sum_{i=1}^{n}{\left[{\bar{\phi }}_{i}\left(y\right)-\phi_{i}\right]}^{2}\cdot f\left(y\right)dy \end{array}$$

(15) $$\begin{array}{cc} \; & =E\left[\frac{1}{n}\sum_{i=1}^{n}{\left[{\bar{\phi }}_{i}\left(y\right)-\phi_{i}\right]}^{2}\right], \end{array}$$

where f(y) denotes the distribution of y and *n* is the dimension of the vector ϕ. The CRLB expresses a lower bound for the MSE that the minimum estimated variance is greater than the inverse of the Fisher information matrix I(ϕ) [29],

(16) $$\sigma_{\bar{\phi }}>\sum_{i=1}^{n}{\left({I(\phi )}^{-1}\right)}_{i,i},$$

and the Fisher information matrix can be calculated as

(17) $$\begin{array}{c} I(\phi )=-E\left[\frac{\partial^{2}\log P\left(y,\phi \right)}{\partial \phi^{2}}\right], \end{array}$$

where P(y,ϕ) denotes the likelihood function. We will first calculate the Fisher information matrix under the ideal case that the signal variance is deterministic and noise variance is known. Then, we consider the more general cases when these variances are random or unknown, and finally we will study the effect of multiple measurements.

### 3.1. Ideal Case CRLB

In the ideal case, we assume the noise variance $\sigma^{2}$ is known and the hyperparameter $\Sigma =\mathrm{diag}\left(\alpha \right)=\mathrm{diag}\left(\alpha_{1},\alpha_{2},\ldots ,\alpha_{2K}\right)$, which is associated with the source, is deterministic. Letting unknown parameters vector $\phi ={\left[{\bar{s}}^{T}\;\alpha^{T}\right]}^{T}$, where the incoming source amplitudes $\bar{s}$ is the only random variable. Because unknown parameters ϕ consist of random and deterministic parameters, the hybrid CRLB will be derived in this case. With Bayes’ rule and the assumption that $\sigma^{2}$ is known and Σ is deterministic, the likelihood of the measurement $\bar{y}$ in (3) can be written as

(18) $$\begin{array}{c} P\left(\bar{y},\bar{s};\Sigma ,\sigma^{2}\right)=P\left(\bar{y}|\bar{s},\sigma^{2}\right)P\left(\bar{s}|\Sigma \right), \end{array}$$

where $P(\bar{y}|\bar{s},\sigma^{2})$ is the likelihood of the measurement, and $P(\bar{s}|\Sigma )$ is the prior estimate for the source which appears in the first stage (4) of the hierarchical model. The log likelihood for (18) is written as

(19) $$\begin{array}{c} \log P\left(\bar{y},\bar{s},\Sigma ,\sigma^{2}\right)=\log P\left(\bar{y}|\bar{s},\sigma^{2}\right)+\log P\left(\bar{s}|\Sigma \right). \end{array}$$

With the expression for $P(\bar{y}|\bar{s},\sigma^{2})$ and $P(\bar{s}|\Sigma )$ in [20], (19) can be written as

(20) $$\begin{array}{cc} \log P(\bar{y},\bar{s},\Sigma ,\sigma^{2}) & =-M\log\frac{1}{2\pi }-M\log\left(\sigma^{2}\right)-2K\log\frac{1}{2\pi }\\ \; & \;-\frac{{\left(\bar{y}-\bar{A}\bar{s}\right)}^{T}\left(\bar{y}-\bar{A}\bar{s}\right)}{2\sigma^{2}}-\sum_{i=1}^{2K}\log\sqrt{\alpha_{i}}\\ \; & \;-\sum_{i=1}^{2K}\frac{{\bar{s}}_{i}^{2}}{2\alpha_{i}}, \end{array}$$

where *M* is the number of elements in the receiving array and 2K is the number of entries in $\bar{s}$. The derivative of $\log P(\bar{y},\bar{s},\Sigma ,\sigma^{2})$ with respect to $\bar{s}$ is

(21) $$\frac{\partial \log P(\bar{y},\bar{s},\Sigma ,\sigma^{2})}{\partial \bar{s}}=\frac{{\bar{A}}^{T}\left(\bar{y}-\bar{A}\bar{s}\right)}{\sigma^{2}}-Y\cdot \bar{s},$$

where $Y\in R^{2K\times 2K}$ is a diagonal matrix with $Y_{\left(i,i\right)}=\frac{1}{\alpha_{i}}$ and the corresponding second derivative is

(22) $$\frac{\partial^{2}\log P\left(\bar{y},\bar{s},\Sigma ,\sigma^{2}\right)}{\partial {\bar{s}}^{2}}=-\frac{{\bar{A}}^{T}\bar{A}}{\sigma^{2}}-Y.$$

Similarly we can calculate the derivative of $\log P(\bar{y},\bar{s},\Sigma ,\sigma^{2})$ with respect to $\alpha_{i}$, which is

(23) $$\frac{\partial \log P(\bar{y},\bar{s},\Sigma ,\sigma^{2})}{\partial \alpha_{i}}=-\frac{1}{2\alpha_{i}}+\frac{s_{i}^{2}}{2\alpha_{i}^{2}},$$

and the second derivatives are

(24) $$\frac{\partial^{2}\log P\left(\bar{y},\bar{s},\Sigma ,\sigma^{2}\right)}{\partial \alpha_{i}^{2}}=\frac{1}{2\alpha_{i}^{2}}-\frac{s_{i}^{2}}{\alpha_{i}^{3}},$$

(25) $$\frac{\partial^{2}\log P\left(\bar{y},\bar{s},\Sigma ,\sigma^{2}\right)}{\partial \alpha_{i}\partial s_{i}}=\frac{s_{i}}{\alpha_{i}^{2}}.$$

I(ϕ) is calculated by using (17),

(26) $$I\left(\phi \right)=\left[\begin{array}{cc} I(\bar{s}) & I(\bar{s},\Sigma )\\ I{\left(\bar{s},\Sigma \right)}^{T} & I(\Sigma ) \end{array}\right],$$

where

$$\begin{array}{cc} I(\bar{s}) & =-E_{\phi }\left\{\frac{\partial^{2}\log P\left(\bar{y},\bar{s},\Sigma ,\sigma^{2}\right)}{\partial {\bar{s}}^{2}}\right\}\in R^{2K\times 2K},\\ I(\bar{s},\Sigma ) & =-E_{\phi }\left\{\frac{\partial^{2}\log P\left(\bar{y},\bar{s},\Sigma ,\Sigma^{2}\right)}{\partial \bar{s}\partial \Sigma }\right\}\in R^{2K\times 2K},\\ I(\Sigma ) & =-E_{\phi }\left\{\frac{\partial^{2}\log P\left(\bar{y},\bar{s},\Sigma ,\sigma^{2}\right)}{\partial \Sigma^{2}}\right\}\in R^{2K\times 2K}. \end{array}$$

From (22), (24) and (25), we can have

(27) $$I\left(\phi \right)=\left[\begin{array}{cc} \frac{{\bar{A}}^{T}\bar{A}}{\sigma^{2}}+Y & 0\\ 0 & \xi \end{array}\right],$$

where $\xi \in R^{2K\times 2K}$ is a diagonal matrix with $\xi_{\left(i,i\right)}=-E\left(\frac{1}{2\alpha_{i}^{2}}-\frac{s_{i}^{2}}{\alpha_{i}^{3}}\right)=\frac{1}{2\alpha_{i}^{2}}$. From (27), it can be seen the Fisher matrix depends on the choice of sampling grid (i.e., $\bar{A}$), measurement conditions (i.e., $\sigma^{2}$) and the hyperparameters. The CRLB of the estimated variance for the unknown variable is proportional to the corresponding diagonal elements of the inverse of the Fisher’s matrix. However, it is not very convenient to write the expression of the diagonal elements of the inverse of the first block in (27), if we have a look at the diagonal elements of the block, it is clear that they will increase as the number of antennas increases and as $\sigma^{2}$ and α become smaller. The lower bounds are also proportional to the reciprocal of the diagonal elements of the Fisher’s matrix [29]. Therefore, it can be concluded that the CRLB decreases as the number of antennas increases and as $\sigma^{2}$ and α become smaller in this case.

### 3.2. Bayesian CRLB

We now consider the second case where Σ is also random and the Bayesian CRLB will be derived. Letting $\phi ={\left[{\bar{s}}^{T}\;\alpha^{T}\right]}^{T}$, Σ is drawn from the prior distribution which is defined in (5), so when calculating the Fisher information matrix, the prior distribution should be considered. The second derivatives of the log likelihood with respect to the unknown parameters are same as the first case ((22), (24), (25)), and each block of the Fisher information matrix becomes

(28) $$\begin{array}{c} I\left(\bar{s}\right)=\frac{{\bar{A}}^{T}\bar{A}}{\sigma^{2}}+\kappa , \end{array}$$

(29) $$\begin{array}{c} I(\bar{s},\Sigma )=0, \end{array}$$

(30) $$\begin{array}{c} I(\Sigma )=\omega , \end{array}$$

where we reuse the same notations in the first case for the Fisher information matrix, κ and ω are both diagonal matrices with $\kappa_{\left(i,i\right)}=E\left(\frac{1}{\alpha_{i}}\right)$ and $\omega_{\left(i,i\right)}=E\left(\frac{1}{2\alpha_{i}^{2}}\right)$. By using Jensen’s inequality and Equation (5), we can have

(31) $$\begin{array}{c} E\left(\frac{1}{\alpha_{i}}\right)\geq \frac{1}{E(\alpha_{i})}=1, \end{array}$$

(32) $$\begin{array}{c} E\left(\frac{1}{2\alpha_{i}^{2}}\right)\geq \frac{1}{2E(\alpha_{i}^{2})}=\frac{\lambda }{2}. \end{array}$$

The Fisher information matrix is written as

(33) $$I\left(\phi \right)⪰\left[\begin{array}{cc} \frac{{\bar{A}}^{T}\bar{A}}{\sigma^{2}}+I & 0\\ 0 & \frac{\lambda }{2}I \end{array}\right].$$

In this case, the Fisher information matrix can be computed without knowing the realization of Σ. Now instead of deriving the lower limit for the incoming source directly by Bayesian CRLB, we notice that the dependence of the source on the hyperparameter λ can be characterized by marginalizing out Σ, which would then be seen as a nuisance parameter:

(34) $$\begin{array}{c} P\left(\bar{s}|\lambda \right)=\prod_{i=1}^{2K}\int_{0}^{\infty }P\left({\bar{s}}_{i}|\alpha_{i}\right)P\left(\alpha_{i}|\lambda \right)d\alpha_{i}. \end{array}$$

By using ((4) and (5)) and the identity $\int_{0}^{\infty }\exp\left(-\frac{s_{i}^{2}}{2a_{i}}-\frac{\lambda a_{i}}{2}\right)da_{i}=\frac{\sqrt{\pi }}{\sqrt{2\lambda }}\exp\left(-\sqrt{\lambda }\left|s_{i}\right|\right)$, we see that the distribution of the source is Laplacian:

(35) $$\begin{array}{c} P\left(\bar{s}|\lambda \right)=\frac{\lambda^{K}}{2^{2K}}\exp\left(-\sqrt{\lambda }\sum_{i=1}^{2K}\left|{\bar{s}}_{i}\right|\right). \end{array}$$

Now, the unknown parameters become $\bar{s}$ and λ. The vector of unknown parameters is therefore written as $\phi ={\left[{\bar{s}}^{T}\;\lambda \right]}^{T}$. The Fisher information matrix I(ϕ) therefore consists of four blocks,

(36) $$I\left(\phi \right)=\left[\begin{array}{cc} I(\bar{s}) & I(\bar{s},\lambda )\\ I{\left(\bar{s},\lambda \right)}^{T} & I(\lambda ) \end{array}\right],$$

where

$$\begin{array}{cc} I(\bar{s}) & =-E_{\phi }\left\{\frac{\partial^{2}\log P\left(\bar{y},\bar{s},\sigma^{2};\lambda \right)}{\partial {\bar{s}}^{2}}\right\}\in R^{2K\times 2K},\\ I(\bar{s},\lambda ) & =-E_{\phi }\left\{\frac{\partial^{2}\log P\left(\bar{y},\bar{s},\sigma^{2};\lambda \right)}{\partial \bar{s}\partial \lambda }\right\}\in R^{2K\times 1},\\ I(\lambda ) & =-E_{\phi }\left\{\frac{\partial^{2}\log P\left(\bar{y},\bar{s},\sigma^{2};\lambda \right)}{\partial \lambda^{2}}\right\}. \end{array}$$

The joint distribution $P(\bar{y},\bar{s},\sigma^{2};\lambda )$ is obtained by combining the likelihood and the prior distribution, $P\left(\bar{y},\bar{s},\sigma^{2};\lambda \right)\propto P\left(\bar{y}|\bar{s},\sigma^{2}\right)P\left(\bar{s}|\lambda \right)P\left(\lambda \right)$. With (35) and ignoring the constant terms, the log likelihood of the joint distribution is

(37) $$\begin{array}{cc} \log P(\bar{y},\bar{s},\sigma^{2};\lambda ) & =-\frac{{\left(\bar{y}-\bar{A}\bar{s}\right)}^{T}\left(\bar{y}-\bar{A}\bar{s}\right)}{2\sigma^{2}}\\ \; & \;+\left(K+1\right)\log\lambda -\sqrt{\lambda }\sum_{i=1}^{2K}\left|{\bar{s}}_{i}\right|, \end{array}$$

and the first derivative of the log likelihood $P(\bar{y},\bar{s},\sigma^{2};\lambda )$ with respect to $\bar{s}$ is

(38) $$\frac{\partial \log P(\bar{y},\bar{s},\sigma^{2};\lambda )}{\partial {\bar{s}}_{i}}=\left\{\begin{array}{cc} \frac{{{\bar{A}}_{i}}^{T}\left(y-{\bar{A}}_{i}\bar{s}\right)}{\sigma^{2}}-\sqrt{\lambda }, & {\bar{s}}_{i}\geq 0,\\ \frac{{{\bar{A}}_{i}}^{T}\left(y-{\bar{A}}_{i}\bar{s}\right)}{\sigma^{2}}+\sqrt{\lambda }, & {\bar{s}}_{i}<0. \end{array}\right.$$

We continue to obtain the second derivative of $\log P(\bar{y},\bar{s},\sigma^{2};\lambda )$ as

(39) $$\frac{\partial^{2}\log P\left(\bar{y},\bar{s},\sigma^{2};\lambda \right)}{\partial {\bar{s}}^{2}}=-\frac{{\bar{A}}^{T}\bar{A}}{\sigma^{2}},$$

(40) $$\frac{\partial^{2}\log P\left(\bar{y},\bar{s},\sigma^{2};\lambda \right)}{\partial \bar{s}\partial \lambda }=\left\{\begin{array}{cc} -\frac{1}{2\sqrt{\lambda }}, & {\bar{s}}_{i}\geq 0,\\ \frac{1}{2\sqrt{\lambda }}, & {\bar{s}}_{i}<0. \end{array}\right.$$

From (39), we can calculate $I(\bar{s})$,

(41) $$I(\bar{s})=\frac{{\bar{A}}^{T}\bar{A}}{\sigma^{2}}.$$

Comparing (41) with our original result from (33), a tighter bound for the variance of estimated $\bar{s}$ is derived:

(42) $$I\left(\phi \right)={\left(\frac{{\bar{A}}^{T}\bar{A}}{\sigma^{2}}\right)}^{-1}⪰{\left(\frac{A^{T}A}{\sigma^{2}}+I\right)}^{-1}.$$

In order to obtain the complete Fisher information matrix, the derivatives of $\log P(\bar{y},\bar{s},\sigma^{2};\lambda )$ with respect to λ are required:

(43) $$\begin{array}{cc} \frac{\partial \log P(\bar{y},\bar{s},\sigma^{2};\lambda )}{\partial \lambda } & =\frac{K+1}{\lambda }-\frac{1}{2\sqrt{\lambda }}\sum_{i=1}^{2K}\left|{\bar{s}}_{i}\right|, \end{array}$$

(44) $$\begin{array}{cc} \frac{\partial^{2}\log P\left(\bar{y},\bar{s},\sigma^{2};\lambda \right)}{\partial \lambda^{2}} & =-\frac{K+1}{\lambda^{2}}+\frac{1}{4}\lambda^{-\frac{3}{2}}\sum_{i=1}^{2K}\left|{\bar{s}}_{i}\right|. \end{array}$$

Thus, the Fisher information for λ is given by

(45) $$\begin{array}{cc} I(\lambda ) & =-E\left\{-\frac{K+1}{\lambda^{2}}+\frac{1}{4}\lambda^{-\frac{3}{2}}\sum_{i=1}^{2K}\left|{\bar{s}}_{i}\right|\right\} \end{array}$$

(46) $$\begin{array}{cc} \; & =\frac{K+1}{\lambda^{2}}-\frac{1}{4}\lambda^{-\frac{3}{2}}E\left\{\sum_{i=1}^{2K}\left|{\bar{s}}_{i}\right|\right\}. \end{array}$$

By using the source distribution from (35), we can calculate the expected value of $|s_{i}|$:

(47) $$\begin{array}{cc} E(|s_{i}|) & =\int_{-\infty }^{\infty }\left|s_{i}\right|\cdot p\left(s_{i},\lambda \right)ds_{i} \end{array}$$

(48) $$\begin{array}{cc} \; & =\sqrt{\lambda }\int_{0}^{\infty }s_{i}\exp\left(-\sqrt{\lambda }s_{i}\right)ds_{i}=\frac{1}{\sqrt{\lambda }}. \end{array}$$

Plugging (48) into (46), we have $I\left(\lambda \right)=\frac{K+2}{2\lambda^{2}}$. Then we have the Fisher information matrix

(49) $$I\left(\phi \right)=\left[\begin{array}{cc} \frac{{\bar{A}}^{T}\bar{A}}{\sigma^{2}} & 0\\ 0 & \frac{K+2}{2\lambda^{2}} \end{array}\right],$$

where $I(\bar{s},\lambda )$ is a zero matrix because $s_{i}$ is defined as a normal distributed variable with mean 0 in the first stage and Equation (40) will result in cancellation in the expectation. We see then that the CRLB for the source vector $\bar{s}$ depends only on the matrix $\bar{A}$ and the noise variance $\sigma^{2}$, whereas the CRLB for the hyperparameter λ is proportional on the square of its true value and inversely proportional to the size of the angle grid *K*.

### 3.3. Noise Variance CRLB

We now derive the noise lower bound; we use $\phi =[\bar{s};\sigma^{2}]$ to denote the unknown variables. The Fisher matrix is written as

(50) $$I\left(\phi \right)=\left[\begin{array}{cc} I(\bar{s}) & I(\bar{s},\sigma^{2})\\ I{\left(\bar{s},\sigma^{2}\right)}^{T} & I(\sigma^{2}) \end{array}\right],$$

where $I(\bar{s})$ have been derived in (27) and (49) for situations with the known and the unknown signal prior, and both depend on $\sigma^{2}$. Furthermore, $I(\bar{s},\sigma^{2})$ is a zero matrix, as this pair of variables is assumed to be uncorrelated. Therefore, we will focus on the block $I(\sigma^{2})$; if we denote $\eta =\sigma^{2}$, we can write

(51) $$I\left(\eta \right)=-E\left\{\frac{\partial^{2}\log P\left(\bar{y},\bar{s};\eta \right)}{\partial \eta^{2}}\right\}.$$

The second derivative of the corresponding log likelihood can be written as

(52) $$\frac{\partial^{2}\log P\left(\bar{y},\bar{s};\eta \right)}{\partial \eta^{2}}=-\frac{{\left(\bar{y}-\bar{A}\bar{s}\right)}^{T}\left(\bar{y}-\bar{A}\bar{s}\right)}{\eta^{3}}+\frac{M}{\eta^{2}},$$

where *M* is the number of antenna elements. It is clear that $\bar{y}-\bar{A}\bar{s}=n$, and n has entries which are subject to a Gaussian distribution with mean zero and variance η, we have

(53) $$E\left\{{\left(\bar{y}-\bar{A}\bar{s}\right)}^{T}\left(\bar{y}-\bar{A}\bar{s}\right)\right\}=2M\eta .$$

Therefore we have

(54) $$I\left(\eta \right)=\frac{M}{\eta^{2}},$$

the CRLB of the noise variance is proportional to the square of the true variance and inversely proportional to the number of the antenna elements in the receiver. The complete Fisher information matrix with the known signal prior is

(55) $$I\left(\phi \right)=\left[\begin{array}{cc} \frac{{\bar{A}}^{T}\bar{A}}{\eta }+Y & 0\\ 0 & \frac{M}{\eta^{2}} \end{array}\right],$$

and for the situation with unknown signal prior, we have the Fisher information matrix as

(56) $$I\left(\phi \right)=\left[\begin{array}{cc} \frac{{\bar{A}}^{T}\bar{A}}{\eta } & 0\\ 0 & \frac{M}{\eta^{2}} \end{array}\right].$$

### 3.4. Multiple Measurements

**Figure 4:**
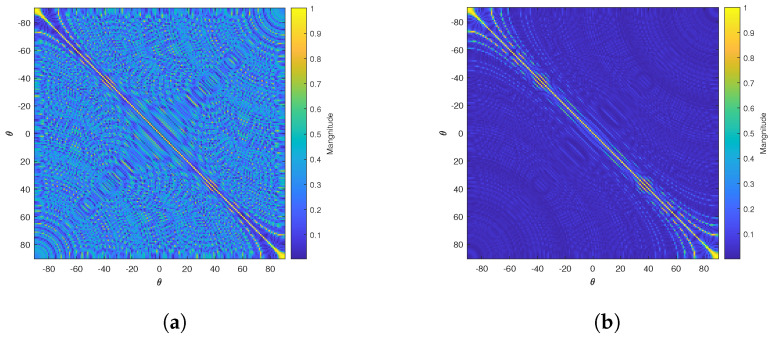
The change of Gram matrix versus the number of measurements. (**a**) Gram matrix with single measurement, M=12. (**b**) Gram matrix with 100 measurements, M=12.

In this subsection, we assume that multiple measurements are available. We are interested in multiple measurements because they can improve the accuracy of the estimated results according to experiments, the improvement can be explained by noting that the effect of noises is mitigated and multiple measurements can reduce the correlation between the columns of the measurement matrix if the measurement matrix changes with time which is common under several scenarios such as distributed ground communication where the locations of receivers could change in each measurement. Thus the difficulty in recovering the source is decreased. For example, the Gram matrices (i.e., $\sum_{l=1}^{L}{\left({\bar{A}}^{l}\right)}^{T}{\bar{A}}^{l}/L$) of single measurement and 100 measurements are presented in Figure 4a,b, in which a 12-element linear array is considered and the location of each receiver is subject to the normal distribution for different measurements.

We can see with multiple measurements, the value of correlation is decreased for different angle. In addition to the change of correlation, CRLB provides us another perspective of studying the effect of multiple measurements to the DoA problem. We can explore the effect of multiple measurements by finding the relationship between the lower bounds with the number of measurements. Two antennas configurations for the CRLB computation are considered here: static and dynamic positions for antenna elements. We start with the static configuration that the element positions are unchanged during the multiple measurements, assuming there are *L* independent measurements and each measurement is denoted by a 2M×1 vector. The DoA problem can be rewritten as Z=AV+n, where $Z\in R^{2M\times L}=\left[y_{1}\;y_{2}\;\ldots \;y_{L}\right]$ denotes the *L* measurements, $A\in R^{2M\times 2K}$ denotes the measurement operator which is unchanged in the static mode, $V\in R^{2K\times L}=\left[s_{1}\;s_{2}\;\ldots \;s_{L}\right]$ denotes the source values for the different measurements, and $N\in R^{2M\times L}$ corresponds to the noise. Because each measurement is assumed to be independent of the others, the likelihood of the *L* measurements can be written as $P\left(Z|X\right)=\prod_{i=1}^{L}P\left(Z_{i}|X_{i}\right)$, where $Z_{i}$ denotes the *i*th measurement and $X_{i}$ denotes the unknown variables at the *i*th snapshot. The Fisher matrix becomes

(57) $$\begin{array}{c} I\left(X\right)=-E\left[\sum_{i=1}^{L}\frac{\partial^{2}P\left(Z_{i}|X_{i}\right)}{\partial X_{i}^{2}}\right]. \end{array}$$

When the incoming sources are assumed to be unchanged, i.e., $X_{1}=X_{2}=\cdot \cdot \cdot =X_{L}$, and then it is easy to see that

(58) $$\begin{array}{c} -E\left[\sum_{i=1}^{L}\frac{\partial^{2}P\left(Z_{i}|X_{i}\right)}{\partial X_{i}^{2}}\right]=-LE\left[\frac{\partial^{2}P\left(Z_{1}|X_{1}\right)}{\partial X_{1}^{2}}\right]. \end{array}$$

With (16) and (58), we can see that the CRLB is reduced by a factor of *L* when compared with the single measurement case in static mode. In contrast, if we consider the dynamic configuration that the element position changes with time, the DoA estimation is rewritten as T=Ws+n, where $T={\left[y_{1}^{T}\;y_{2}^{T}\;\ldots \;y_{L}^{T}\right]}^{T}\in R^{2ML\times 1}$, $W={\left[A_{1}^{T}\;A_{2}^{T}\;\ldots A_{L}^{T}\right]}^{T}\in R^{2ML\times 2K}$, $N={\left[n_{1}^{T}n_{2}^{T}\ldots n_{L}^{T}\right]}^{T}$. Thus, the Fisher information matrix can be written as

(59) $$\begin{array}{c} I\left(X\right)=-E\left[\frac{\partial^{2}P\left(T|X\right)}{\partial X^{2}}\right]. \end{array}$$

Similarly to (41), we can show that

(60) $$E\left[\frac{\partial^{2}P\left(T|X\right)}{\partial X^{2}}\right]\propto W^{T}W.$$

For the DoA estimation problem, $\mathrm{diag}\left(A_{1}^{T}A_{1}\right)=\mathrm{diag}\left(A_{2}^{T}A_{2}\right)=\cdots =\mathrm{diag}\left(A_{L}^{T}A_{L}\right)$, so we can have $\mathrm{diag}\left(W^{T}W\right)=L\cdot \mathrm{diag}\left(A_{n}^{T}A_{n}\right)$, n=1,2,3,⋯L. Because the lower bounds are proportional to the reciprocal of the diagonal elements of the Fisher’s matrix, we obtain an unsurprising conclusion: the minimum estimation variance is reduced by a factor of *L* compared with the single measurement.

## 4. Numerical Results

To test the sharpness of the CRLBs obtained in this paper, we generate numerical results from a simulation involving *L* measurements obtained from an array of *M* elements and a set of *N* incoming sources, the results are obtained by using MATLAB R2021a on a laptop with 8 GB RAM and 1.6 GHz Intel Core i5. The position of the *M* array elements are drawn i.i.d. from the distribution $l_{m}∼U\left[\left(m-1\right)\cdot \Delta s,\left(m-1\right)\cdot \Delta s+r\right]$, where $l_{m}$ denotes the position of the *m*th element, U[a,b] denotes the uniform distribution between *a* and *b*, Δs denotes the distance between the two neighboring antenna elements, and *r* denotes the feasible range for the position of one element. All the results in this section are generated by setting N=3, Δs=24λ and r=6λ, because we are interested in large array spacings.

**Figure 5:**
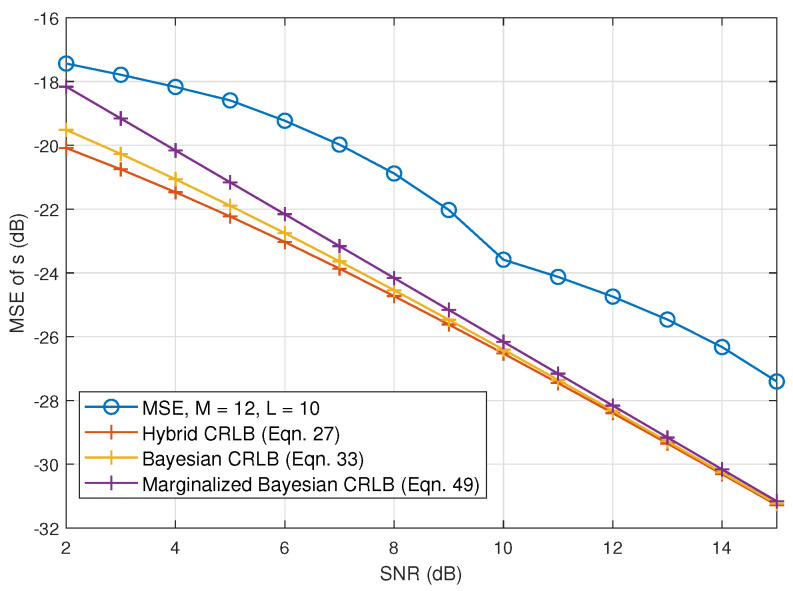
Experimental CRLBs of incoming source estimation as a function of the measurement SNR and M=12, L=10.

Figure 5 shows the derived CRLBs for the source estimator and its numerical MSE as a function of the signal-to-noise ratio (SNR). The magnitude of MSE and CRLBs are converted to dB in order to make the difference more visible in the plot. We can see that under the same condition, marginalized Bayesian CRLB provides the tightest lower bound, especially when SNR is low. When SNR becomes higher, the difference between the three CRLBs becomes smaller. Another point worth noticing is that if we compare the difference between the magnitudes of MSE and CRLBs, the differences actually become smaller with the increase of SNR for all CRLBs; for example for the marginalized Bayesian CRLB, the difference drops from 0.003 at SNR = 2 dB to 0.001 at SNR = 14 dB.

**Figure 6:**
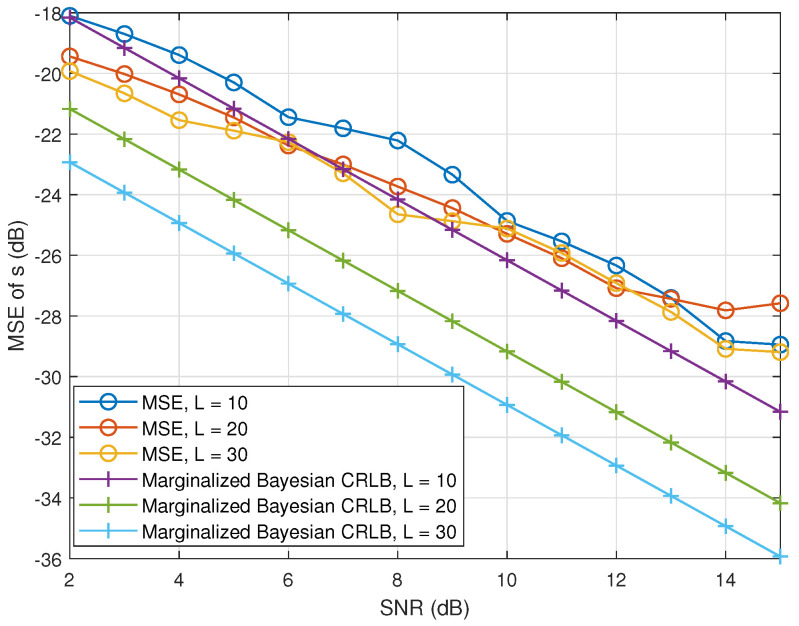
Experimental CRLBs of incoming source estimation as a function of SNR, the number of snapshots and M=12.

Figure 6 shows the marginalized Bayesian CRLB for the source estimator and its numerical MSE as a function of the SNR for several numbers of snapshots *L*. We chose L=10,20, and 30 in the experiment, it is clear that the CRLB decreases as *L* and the SNR increase which explains the effect of multiple snapshots to this DoA estimation method in the perspective of lower bounds. With the increase of SNR, the difference of the MSE and CRLBs decreases for L=20 and 30 in terms of magnitude, and the difference is almost the same for L=10.

**Figure 7:**
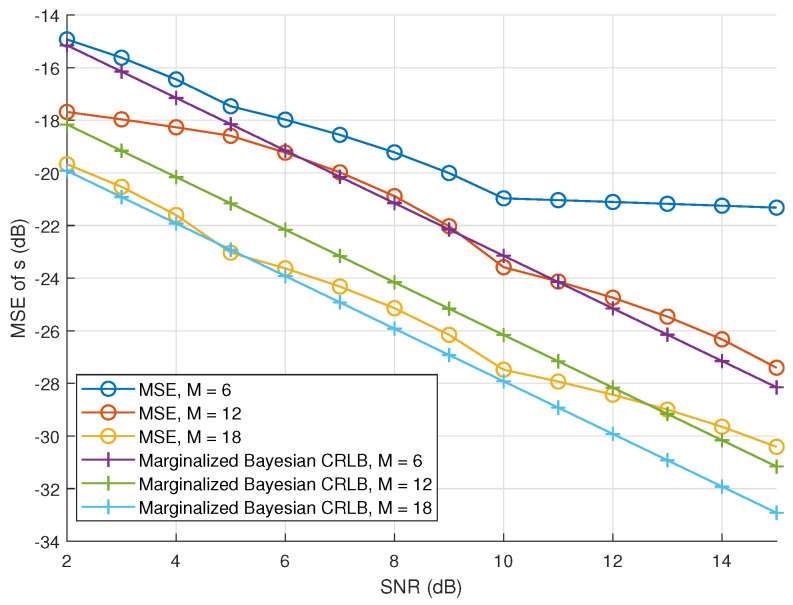
Experimental CRLBs of incoming source estimation as a function of SNR, the number of elements and L=10.

Figure 7 also shows the marginalized Bayesian CRLB and MSE as a function of the SNR for various numbers of receiving elements *M*. This CRLB and MSE decrease as the number of receiving elements increases which verifies the effect of increasing the number of receiving elements to this DoA estimation method. We also notice that as the SNR increases, the difference between MSE and the CRLB becomes larger for the case with M=6, in order to improve the performance of this estimation method in that case, we can try to tune the convergence threshold.

**Figure 8:**
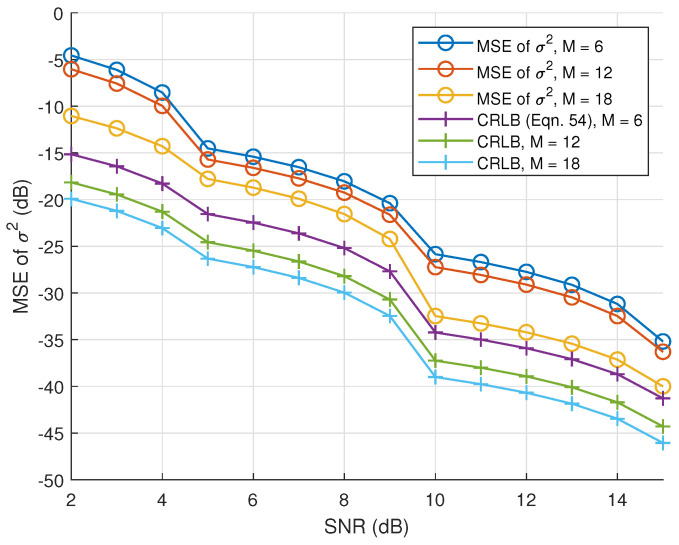
Experimental CRLBs of noise as a function of SNR, the number of elements and L=10.

Finally, Figure 8 also shows the CRLB of the noise variance estimator and its numerical MSE for several numbers of receiving elements *M* under various SNRs. We see that the CRLB decreases as the number of receiving elements increases, which means increasing the number of receivers can also improve the estimation accuracy for the noise, and the difference between the MSE and the CRLB also decreases as SNR increases in terms of magnitude.

## 5. Conclusions

In this paper, we derived the CRLBs of estimated source, hyperparameters and noise in terms of MSE by using the Laplace prior in sparse Bayesian learning and we explored the several factors that can affect the lower bounds. The numerical simulation result is presented for demonstrating the derived lower bounds under different conditions. Among the derived CRLBs, the marginalized Bayesian CRLB is the tightest one. The relationship between the CRLB of the source to be estimated and the number of receiving elements and the number of snapshots is provided in the DoA problem, as we expect, the CRLB decreases as the number of receiving elements and the number of snapshots increase. In the future, we can extend the lower bounds to planar arrays and exploring the lower bounds of off-grid DoA estimation method can be another topic of a future paper. The effect of the convergence threshold is also worth further investigation.

## Data Availability

Not applicable.
